# Diagnostic Performance of Prenatal Ultrasound to Detect Velamentous Cord Insertion in Twin Pregnancies

**DOI:** 10.3390/jcm15083168

**Published:** 2026-04-21

**Authors:** Kodai Minoura, Hiroyuki Tsuda, Yumiko Itoh, Atsuko Tezuka, Tomoko Ando

**Affiliations:** Department of Obstetrics and Gynecology, Japanese Red Cross Aichi Medical Center, Nagoya Daiichi Hospital, Nagoya 453-8551, Japan; kodaim1115@gmail.com (K.M.);

**Keywords:** velamentous cord insertion, diagnostic ultrasound imaging, prenatal diagnosis, twin pregnancy

## Abstract

**Objective:** We aimed to determine the ability of prenatal ultrasound to detect velamentous cord insertion (VCI) in twin pregnancies and identify factors influencing diagnostic sensitivity. **Methods:** This single-center retrospective study included twins delivered between April 2018 and March 2024. We excluded monochorionic monoamniotic twins, those without chorionicity or umbilical cord insertion data, and fetuses that died in utero. Umbilical cord insertion sites assessed by second-trimester transabdominal ultrasound (16 + 0 to 21 + 6 weeks of gestation) using color Doppler imaging were classified as normal, marginal, or velamentous. The results of postnatal macroscopic examinations served as reference standards. We calculated accuracy, sensitivity, specificity, positive (PPV) and negative (NPV) predictive values. The effects of examiner expertise, chorionicity, placental location, ultrasound device, and maternal body mass index (BMI) on diagnostic sensitivity were analyzed in subgroups. **Results:** We confirmed VCI in 45 (8.8%) of 514 delivered fetuses. Prenatal ultrasound correctly identified 14 VCI cases. Sensitivity, specificity, PPV, and NPV were 31.1% (14/45), 98.9% (464/469), 73.7% (14/19), and 93.7% (464/495), respectively. The overall accuracy was 93.0% (478/514). Sensitivity was significantly higher when ultrasound specialists conducted examinations compared with non-specialists and when twins were monochorionic diamniotic twins than dichorionic. Anterior placental location and high-performance ultrasound equipment were also associated with increased sensitivity, but were not statistically significant. Maternal BMI did not affect diagnostic sensitivity. **Conclusions:** Prenatal ultrasonographic detection of VCI in twin pregnancies has high specificity but limited sensitivity. Diagnostic performance was influenced by examiners’ experience and chorionicity. Routine assessment of cord insertion sites and targeted training might improve detection and support the optimized perinatal management of twin pregnancies.

## 1. Introduction

Velamentous cord insertion (VCI) describes when the umbilical cord attaches to fetal membranes rather than directly into the placental mass. Umbilical vessels course through fetal membranes without the protective support of Wharton’s jelly, rendering them susceptible to compression or rupture [1].

Velamentous cord insertion is associated with adverse perinatal outcomes singleton pregnancies, including preterm birth, fetal distress, and premature membrane rupture [2,3,4]. A subset of VCI is complicated by vasa previa, which is a rare serious condition associated with high risk of fetal mortality due to blood loss from ruptured fetal vessels [5,6]. The ranges of VCI incidences are 0.4–11% and 1.6–40% in singleton and twin pregnancies, respectively, and is more prevalent among monochorionic (MC), than dichorionic (DC) twins [3]. The incidences of VCI were 4.4% and 9.7% in DC and monochorionic diamniotic (MCDA) twins, respectively, among 694 twin pregnancies managed at our institution [7].

Velamentous cord insertion is a risk factor for adverse outcomes in singleton pregnancies and it might also contribute to complications during twin pregnancies, including twin-to-twin transfusion syndrome (TTTS), severe birth weight discordance, and fetal death in utero [8,9,10]. However, recent findings of mixed cohorts of DC and MCDA twins have been contradictory, and have associated VCI with vasa previa and placenta accreta spectrum, but without significant links with other adverse perinatal outcomes [11]. We previously highlighted this discrepancy and showed that while VCI was not significantly associated with adverse outcomes in DC twins, it was associated with fetal growth restriction (FGR), TTTS, and preterm birth in MCDA twins [7].

Given its implications for perinatal management, prenatal diagnosis of VCI by ultrasound is important. The reported sensitivity of ultrasound for detecting VCI in singleton pregnancies varies from 25% to 100%, with maximum of 62.5% sensitivity and consistently high specificity [3,12,13,14]. While numerous studies have addressed the association between VCI and various perinatal risks in twin pregnancies, the ability of ultrasound to detect VCI in twin pregnancies has not been specifically investigated.

Therefore, we aimed to determine the diagnostic characteristics of prenatal ultrasound for VCI in twin pregnancies and clarify its role in optimizing perinatal management strategies.

## 2. Methods

### 2.1. Study Design and Population

This single-center, retrospective study of twin pregnancies delivered at our institution was conducted between April 2018 and March 2024. Regarding the sample size, we employed a consecutive sampling method due to the retrospective nature of the study and the relative rarity of VCI. We included all eligible patients who underwent ultrasound assessment of the cord insertion site at our institution. No formal a priori sample size calculation was performed; however, all available data during this period were utilized to maximize the statistical precision of our estimates. The exclusion criteria were monochorionic monoamniotic twin pregnancies, neonates without chorionicity information, neonates without information about umbilical cord insertion sites, and intrauterine fetal death.

This study complied with the ethical principles enshrined in the Declaration of Helsinki (2024 amendment) and was approved by the Ethics Committee at the Japanese Red Cross Aichi Medical Center, Nagoya Daiichi Hospital, Nagoya, Japan (Approval No: 2024-099, Approval date: 10 September 2024). The need for informed consent was waived as we investigated innominate data.

### 2.2. Pregnancy Management

When a twin pregnancy was confirmed, chorionicity and amnionicity were assessed during the first trimester using ultrasound. Specifically, a “twin peak” (or “lambda”) sign between inter-twin membranes indicated a DC pregnancy, whereas a “T” sign suggested an MCDA pregnancy [15]. We examined MC twins every 2 weeks from 16 weeks of gestation, and DC twins, every 4 weeks until 22 weeks and every 2 weeks afterwards.

### 2.3. Data Collection and Definition

Maternal and fetal data were extracted from medical records. We categorized conception as being spontaneous, induced, or by assisted reproductive technology (ART). Umbilical cord insertion sites and placental locations were determined by transabdominal ultrasound during the second trimester (between gestational weeks 16 + 0 and 21 + 6) [16,17]. Umbilical cord insertion was not routinely diagnosed by ultrasound at our institution. Therefore, pregnancies without a cord insertion diagnosis during this period, and pregnant women who first presented at our institution after this period, were excluded.

We evaluated umbilical cord insertion sites in twin fetuses using transabdominal ultrasound device with color Doppler imaging to visualize vascular structures. Cord attachments were categorized as follows: (1) normal, if inserted directly into the placental parenchyma; (2) marginal, if inserted within 3 cm of the margin of placental parenchyma; and (3) velamentous (VCI), if the cord inserted into the fetal membranes with vessels running unprotected along the membrane surface before reaching the placenta. At the time of delivery, labels were attached to the umbilical cord of the presenting twin to distinguish between the two cord insertion sites for further macroscopic investigation. Midwives macroscopically examined postpartum umbilical cords to document marginal insertion sites within 3 cm from the margin of the placental parenchyma and those attached to the membranes and vessels reaching the placental parenchyma by traversing fetal membranes classified as VCI [14,18].

Placental locations were categorized as anterior, posterior, or fundal. For the subgroup analysis of diagnostic sensitivity according to placental location, fundal placentas were reclassified as either anterior or posterior. This reclassification was based on a retrospective review of the ultrasound findings to determine the predominant orientation of the placental disc and the cord insertion site. This approach was adopted to evaluate how the physical accessibility of the placenta—with anterior locations generally offering superior visibility compared to posterior ones—impacts the examiner’s ability to detect VCI. We re-evaluated images acquired using Voluson S6, E8, and S8 ultrasound systems (General Electric Medical Systems, Milwaukee, WI, USA). Placental locations were reclassified as anterior or posterior according to the predominant distribution of the placental parenchyma between April 2018 and October 2022, April 2018 to December 2020, and January 2021 until the present day.

We defined ultrasound specialists as physicians who were certified by the Japan Society of Ultra Sonics in Medicine and had >20 years of experience in obstetric ultrasound and compared them with other physicians. The present study included one specialist (H.T.), three and 24 obstetricians with 10–15 and <10 years of experience in obstetric ultrasound, respectively.

### 2.4. Statistical Analysis

All data were statistically analyzed using R v. 4.4.1. We analyzed subgroups comprising body mass index (BMI), placental location, chorionicity, diagnostic expertise, of physicians and ultrasound devices. 95% confidence intervals (CIs) for all diagnostic performance metrics were calculated using the Clopper-Pearson method. The sensitivity of VCI detected using ultrasound was compared using chi-squared tests. Multiple tests were adjusted using the Bonferroni correction.

## 3. Results

**Figure 1:**
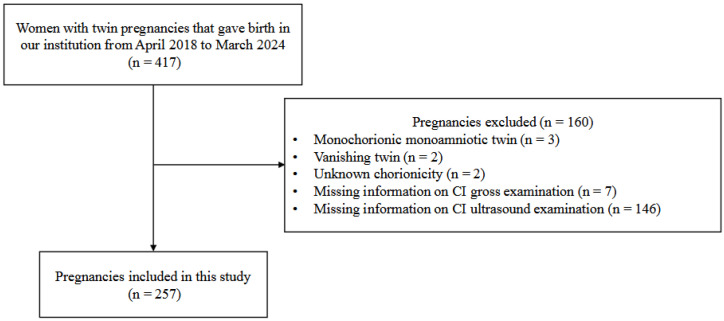
Flow chart of study population.

Figure 1 shows the flow of patients through the study. We identified VCI in 45 (8.8%) of 514 fetuses, which was consistent with reported rates among twin pregnancies. Table 1 and Table 2 summarize maternal and fetal characteristics. We found that DC twins were significantly more frequent when pregnancies involved ARTs (Table 1). Placentae located in the posterior wall of the uterus were significantly more frequent in MCDA twins (Table 2). At our institution, MCDA twin pregnancies, which are considered high risk, are more frequently screened by ultrasound specialists. Consequently, the proportions of cord insertion sites evaluated by a specialist were significantly higher among MCDA, than DC twin pregnancies. Maternal BMI, cord insertion site, and placental location did not significantly differ between MCDA and DC twins.

We assessed 417 twin pregnancies delivered at our institution between April 2018 and March 2024. Exclusion criteria were monochorionic monoamniotic twin pregnancies (n = 3), vanishing twin cases (n = 2), unknown chorionicity (n = 2), missing information on cord insertion during postnatal gross examination (n = 7), and missing information on cord insertion on prenatal ultrasound (n = 146). Ultimately, 257 pregnancies were included in this study.

We prenatally diagnosed 14 of the 45 VCIs that were confirmed by gross examination. The sensitivity, specificity, PPV, and NPV for VCI detection were 31.1% (14/45; 95% CI: 18.2–46.6%), 98.9% (464/469; 95% CI: 97.5–99.7%), 73.7% (14/19; 95% CI: 48.8–90.9%), and 93.7% (464/495; 95% CI: 91.2–95.7%), respectively. The overall accuracy was 93.0% (478/514; 95% CI: 90.4–95.0%).

Because VCI is relatively rare, ultrasound screening revealed high specificity and NPV, which was consistent with previous findings among singletons [3]. Among 31 false-negative insertions, seven were marginal, 21 were normal, and three sites were difficult to identify where the umbilical cords were inserted. Five false-positive comprised one marginal and four normal insertions.

Factors that potentially influenced the diagnostic performance of VCI detection, comprised examiner expertise, chorionicity, placental location, ultrasound device type, and maternal BMI. Given the screening nature of the examination, we focused on sensitivity for intergroup comparisons (Table 3). Sensitivity was significantly higher when VCI was diagnosed by ultrasound specialists compared with non-specialists (*p* < 0.05). Sensitivity was significantly lower in DC, than MCDA twins (*p* < 0.01). The univariate logistic analysis revealed that the odds of detecting VCI were significantly increased when the examination was performed by a specialist (OR: 5.81; 95% CI: 1.30–26.0) and in cases of MCDA pregnancies (OR: 10.9; 95% CI: 2.03–58.6).

Although not statistically significant, sensitivity was increased when high-end ultrasound devices were applied or when placentae were located on the anterior uterine wall, which was consistent with clinical findings (*p* = 0.523 and 0.150, respectively). Maternal BMI did not significantly affect diagnostic sensitivity.

## 4. Discussion

To the best of our knowledge, this study is the first to evaluate the diagnostic performance of prenatal ultrasound examinations for VCI focued on twin pregnancies. We previously reported that VCI in MCDA twins is a significant risk factor for various adverse perinatal outcomes. Therefore, prenatal identification of VCI is essential for optimizing perinatal management during twin gestations. TTTS, FGR, and preterm labor are associated with VCI, and these conditions often progress rapidly. Therefore, twin pregnancies require careful and intensive surveillance and establishing an optimal management schedule should be an important area for future research.

In the present study, the sensitivity for detecting VCI was 31.1% in the present study, which was lower than that of a study with the highest quality of data among singleton pregnancies [17]. This reduced sensitivity might be attributable to the inherent anatomical and technical complexities of ultrasound examination in twin pregnancies. This suggests that greater vigilance is required when evaluating cord insertion in such circumstances.

Interestingly, umbilical cord insertion sites were undetectable in 9.6% (3 out of 31) of false-negative cases, a rate higher than the overall rate of 1.6% (8 out of 514). Therefore, examiners should exercise caution and suspect potential VCI when the cord insertion site cannot be clearly visualized. The subgroup analysis revealed that experienced physicians achieved significantly higher sensitivity, indicating that adequate training and experience are key determinants of diagnostic performance. Given the diagnostic challenges implied by low overall sensitivity, the need for structured education for examiners should be emphasized. Furthermore, because many institutions might not routinely assess placental cord insertion sites, awareness of the clinical significance of VCI and efforts to standardize its evaluation are necessary. Our research underscores the imperative need for standardized screening protocols and structured education to improve the detection rates of VCI. Such systematic approaches are essential for translating diagnostic findings into better clinical outcomes and perinatal safety.

The diagnostic sensitivity was significantly lower in DC, than in MCDA twins. This could be explained by the more complex intrauterine structures of DC twins, which have two separate placentas and, thus, a more challenging sonographic environment for identifying cord insertions. Moreover, VCI was more frequently detected when the placenta was located on the anterior, than on the posterior uterine wall, because fetal structures are less likely to be obstacles when detecting the cord insertion sites. This finding is consistent with our clinical experience and previous studies of singleton pregnancies [17].

High-performance ultrasound equipment might further enhance the diagnostic accuracy of VCI. Although the International Society of Ultrasound in Obstetrics and Gynecology guidelines specify the minimum technical requirements for obstetric ultrasound, advanced imaging devices could provide additional diagnostic advantages and support clinicians in detecting VCI more reliably [19].

While prenatal ultrasound remains the only feasible and non-invasive modality for screening VCI, our findings highlight its significant diagnostic limitations. The low sensitivity observed indicates that a ‘normal’ ultrasound finding does not definitively rule out VCI. Given that missed VCI cases carry substantial perinatal risks, clinical management during labor must remain proactive. Clinicians should not rely solely on prenatal ultrasound findings and must maintain a high index of suspicion for VCI-related complications.

## 5. Strengths

To the best of our knowledge, this is the first study to determine the diagnostic characteristics of VCI using ultrasound in twin pregnancies. Furthermore, subgroup analyses identified factors that influence the diagnostic accuracy of VCI. The study included 45 VCIs among 514 fetuses, which was a comparable sample size to the most robust datasets generated from singleton pregnancies.

## 6. Limitations

As VCI is relatively rare, the number of placentae was insufficient to conduct multivariate analysis to avoid confounding results. For example, the ultrasound equipment used in our institution has become increasingly advanced over time, the skills of examiners have also improved. Therefore, to completely disentangle the extent to which each factor independently contributes to the observed improvement in diagnostic sensitivity is challenging. Also, MCDA cases were more frequently examined by specialists due to their higher clinical risk, which may lead to potential bias. As data regarding twin pregnancies will accumulate in the future, analyses will likely become more detailed and comprehensive.

Furthermore, we must acknowledge the potential for selection bias due to missing data since systematic screening and documentation of the umbilical cord insertion site were not strictly mandated for every case.

### Clinical Implications

Previous studies suggest that prenatal diagnosis of VCI contributes to improved perinatal management in twin pregnancies. Our results indicate that VCI detection is more challenging in twins than in singletons, emphasizing the need for greater vigilance during umbilical cord insertion screening in twins. Furthermore, our subgroup analysis identified several factors influencing diagnostic sensitivity, clarifying the specific characteristics of ultrasound diagnosis for VCI in twins.

## 7. Conclusions

Prenatal ultrasonographic diagnosis of VCI in twin pregnancies remains challenging, with lower sensitivity than that in singleton pregnancies. Diagnostic performance is influenced by the experience of examiners, chorionicity, and placental location. Training programs to improve sonographic expertise and routine assessment of cord insertion sites should be encouraged to enhance diagnostic accuracy. Furthermore, high-performance ultrasound equipment might help to detect VCI and contribute to improved perinatal management of twin pregnancies.

## Figures and Tables

**Table 1:** Characteristics of twin pregnancies.

	MCDA (n = 104)	DC (n = 153)	*p*-Value
Age (y)	32 (17–42)	33 (20–44)	0.102
BMI (kg/m^2^)	21.2 (3.1)	21.4 (3.2)	0.641
ART	30 (28.8%)	101 (66.0%)	<0.001
Nulliparity	52 (50.0%)	97 (63.4%)	0.045

Data are presented as medians (minimum–maximum), means (standard deviation), or n (%). ART, assisted reproductive technology; BMI, body mass index; DC, dichorionic; MCDA, monochorionic diamniotic. Statistical tests were performed with chi-squared tests.

**Table 2:** Characteristics of fetuses.

	MCDA (n = 208)	DC (n = 306)	*p*-Value
Cord insertion site			0.531
Normal	154 (74.1%)	242 (79.1%)	
Marginal	31 (14.9%)	42 (13.7%)	
Velamentous	23 (11.1%)	22 (7.2%)	
Placental location			<0.01
Anterior	91 (43.8%)	153 (50.0%)	
Posterior	19 (9.1%)	8 (2.6%)	<0.01
Fundal	92 (44.2%)	133 (43.5%)	
Unspecified	6 (2.9%)	12 (3.9%)	

Data are presented as n (%). DC, dichorionic; MCDA, monochorionic diamniotic. Statistical tests were performed with chi-squared tests. Multiple tests were adjusted using the Bonferroni correction.

**Table 3:** Subgroup analysis of VCI detection by ultrasonography.

	Sensitivity (%)	*p*
Examiner expertise		<0.05
Specialist	11/23 (47.8%)	
Else	3/22 (13.6%)	
Chorionicity		<0.01
MCDA	12/23 (52.2%)	
DC	2/22 (9.1%)	
Placental location		0.150
Anterior	9/22 (40.9%)	
Posterior	3/19 (15.8%)	
Ultrasonography device		0.523
Voluson S6	3/15 (20.0%)	
Voluson S8	7/19 (36.8%)	
Voluson E8	4/11 (36.4%)	
BMI (kg/m^2^)		0.623
Low (<18)	0/2 (0.0%)	
Normal (18–25)	12/37 (32.4%)	
High (>25)	2/6 (33.3%)	

Data are shown as n/N (%). BMI, body mass index; DC, dichorionic; MCDA, monochorionic diamniotic; VCI, velamentous cord insertion. Statistical tests were performed with chi-squared tests. Multiple tests were adjusted using the Bonferroni correction.

## Data Availability

The data that support the findings of this study are available from the corresponding author upon reasonable request (Email: hirotty7099@yahoo.co.jp).
